# Neurological Immunotoxicity from Cancer Treatment

**DOI:** 10.3390/ijms22136716

**Published:** 2021-06-23

**Authors:** Sarah F. Wesley, Aya Haggiagi, Kiran T. Thakur, Philip L. De Jager

**Affiliations:** 1 Multiple Sclerosis Center, Department of Neurology, Division of Neuroimmunology, Columbia University Vagelos College of Physicians and Surgeons, Columbia University Irving Medical Center-New York Presbyterian Hospital, 710 W 168th Street, Neurological Institute, New York, NY 10032, USA; pld2115@cumc.columbia.edu; 2 Department of Neurology, Division of Neuro-Oncology, Columbia University Vagelos College of Physicians and Surgeons, Herbert Irving Comprehensive Cancer Center, New York-Presbyterian Hospital, New York, NY 10032, USA; amh2237@cumc.columbia.edu; 3 Program in Neuroinfectious Diseases and Related Disorders, Division of Critical Care and Hospitalist Neurology, Columbia University Irving Medical Center-New York Presbyterian Hospital, New York, NY 10032, USA; ktt2115@cumc.columbia.edu

**Keywords:** neurotoxicity, immune-related adverse events, checkpoint inhibitors, chimeric antigen receptor T-cell therapies

## Abstract

The emergence of immune-based treatments for cancer has led to a growing field dedicated to understanding and managing iatrogenic immunotoxicities that arise from these agents. Immune-related adverse events (irAEs) can develop as isolated events or as toxicities affecting multiple body systems. In particular, this review details the neurological irAEs from immune checkpoint inhibitors (ICI) and chimeric antigen receptor (CAR) T cell immunotherapies. The recognition and treatment of neurological irAEs has variable success, depending on the severity and nature of the neurological involvement. Understanding the involved mechanisms, predicting those at higher risk for irAEs, and establishing safety parameters for resuming cancer immunotherapies after irAEs are all important fields of ongoing research.

## 1. Introduction

Iatrogenic neuroinflammatory diseases are a growing subfield of neuroimmunology and relates to cancer immunotherapy, and thus to oncology. These differing conditions can at times appear to be clinically and radiographically indistinct from more classical neuroinflammatory diseases, such as multiple sclerosis or myasthenia gravis; however, they are distinguished by a history of exposure to immunomodulation as well as differences in management relative to sporadic neuroinflammatory disease.

In particular, immunotherapies used for the treatment of cancer are becoming more prevalent and have inaugurated an entire field dedicated to understanding iatrogenic immunotoxicity. While there are several types of immunotherapies being used to treat cancer, many are still in the early stages of development. Current FDA-approved mechanisms include immune checkpoint inhibitors (ICI) [1] and chimeric antigen receptor (CAR) T cell therapies [2], whose mechanisms will be discussed in further detail later in this review.

While the treatment of cancer has been revolutionized by many of these therapies, people with autoimmune conditions were largely excluded from clinical trials over concerns for immunological side effects. Immune-related adverse events (irAEs) observed with cancer immunotherapies are common, and those involving the nervous system are often of significant concern, given a propensity for higher grades of toxicity, which may be refractory to standard management strategies [3,4]. Manifestations of irAEs include both worsening of existing autoimmune conditions, such as multiple sclerosis [5,6,7], or de novo neurological conditions, such as encephalitis, in a previously neurologically non-impaired individual [8,9,10].

The aim of this review is to describe the mechanisms, clinical presentations, and management of neurological irAEs from cancer immunotherapies. This review will specifically focus on neurological irAEs from currently available, FDA-approved cancer immunotherapies: ICIs and CAR T cell therapy.

## 2. Immune Checkpoint Inhibitors

### 2.1. Indications

ICIs are increasingly used as monoclonal antibodies that target the regulatory pathways involved in the immune response to cancer, allowing immune recognition and destruction of the tumor. Their therapeutic function is based on the naïve T cells needed for a second signal or “two signal model” for activation.

After interaction between the tumor antigen (Ag) and T cell receptor (TCR), costimulatory receptors on the T cell then interact with ligands on an antigen-presenting cell (APC) or tumor cell to allow T cell activation. Costimulatory receptors are upregulated when T cells are activated, and inhibitory receptors limit overstimulation of the immune response after an encounter with Ag. Co-receptors that act as negative modulators of immune response include cytotoxic T-lymphocyte-associated protein 4 (CTLA-4), programmed cell death protein 1 (PD-1), T cell immunoreceptor with immunoglobulin and ITIM domains (TIGIT), and lymphocyte activation gene-3 (LAG3).

**Figure 1:**
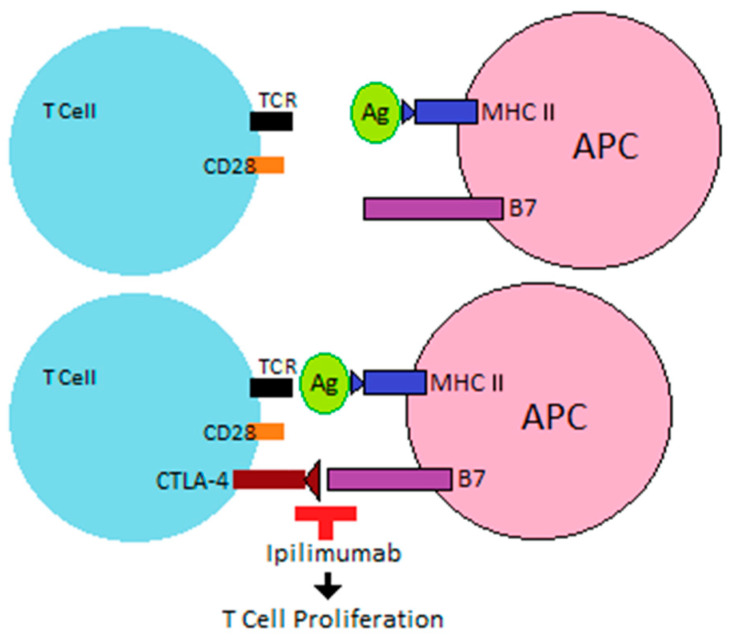
Ipilimumab prevents negative T cell co-receptor CTLA-4 interaction with B7 on APCs, allowing for T cell proliferation.

T cell activation occurs when an APC presents tumor Ag via major histocompatibility complex (MHC) to the TCR, leading to interaction between costimulatory receptor CD28 on the T cell and B7 (specifically CD80 and CD86) on an APC. The appearance of CTLA-4 on the T cell, which has a higher affinity than CD28 for CD80 and CD86, leads to T cell inactivation [11,12]. By blocking this interaction, monoclonal antibodies, such as ipilimumab, allow for continued T cell proliferation (Figure 1).

PD-1 is expressed on activated T cells several hours later than CTLA-4 and is observed on T cells expressing an “exhausted” phenotype from chronic stimulation, thus limiting the immune response of such cells. It also induces expression of programmed cell death protein 1 ligand (PD-L1) on the tumor cell, binds PD-L1 and PD-L2, and causes downregulation of T cell activity [12]. Anti-PD-1 and anti-PD-L1 monoclonal antibodies prohibit this inhibitory mechanism, allowing for T cell proliferation and destruction of tumor cells.

The current FDA-approved targets for ICIs are PD-1, PD-L1, and CTLA-4 (Table 1). With more FDA approvals for usage of these medications and with growing interest in their use, the use of ICIs will to continue to grow. Other biologics in the pipeline are antibodies against TIGIT and LAG3, and many trials focus on using a combination of biological therapy with concurrent ICIs.

### 2.2. Mechanisms of ICI-Related Immunotoxicity

While these agents have revolutionized cancer treatment, ICI mechanisms can lead to a loss of immune regulation, thus raising concern for de novo inflammatory disease or worsening of preexisting disease [13]. There is growing knowledge of the mechanisms involved in toxicity, but many aspects remain poorly understood. Heterogeneity in types of irAEs, the target organ, as well as the variable timing of onset of reactions after exposure suggest that there are different mechanisms involved amongst the various types of ICIs, as well as inherent differences in host susceptibility. For example, some reactions occur shortly after a single dose and others are delayed by many months after finishing a longer treatment course.

#### 2.2.1. Implications of CTLA-4 Blockade

CTLA-4 is a molecule expressed on T cells after interaction with Ag, which competes with costimulatory molecule CD28 for binding to CD80 and CD86; moreover, CTLA-4 also appears on Foxp3+ regulatory T cells (T_reg_). Blockade of CTLA-4 raises concern for unregulated T lymphocyte activation and proliferation with concurrent loss of T_reg_ functioning.

Animal models have shown that germ-line deficiency of CTLA-4 in mice leads to extensive T cell lymphoproliferative disease, which is rapidly fatal, as well as autoimmunity. Acquired CTLA-4 deficiency in mice also leads to autoimmunity in several organ systems, although it does not appear to be as fatal as congenital lack of CTLA-4. Klocke et al. studied experimental autoimmune encephalitis (EAE) in mice in order to understand CTLA-4’s role in peripheral tolerance. Interestingly, CTLA-4 knockout mice had protection against myelin oligodendrocyte glycoprotein_79–96_ (MOG_79–96_) peptide-induced EAE, but mice injected with MOG_1–125_ protein to induce a more B cell-dependent process went on to still develop EAE. This suggests that CTLA-4 also likely has a regulatory effect on B cell responses [14].

Other studies in mice have shown impaired T_reg_ function and decreased survival, along with an increase in effector T cell numbers. In human trials of anti-CTLA-4 antibody ipilimumab, however, there are conflicting findings. The absolute number of T_reg_ have been found to be low or unchanged across studies [15,16], although the relationship between numbers of and function of T_reg_ is complex. CTLA-4 blockade has also been shown to increase in circulating type 17 T helper cells (T_H_17), which produce IL-17, a proinflammatory cytokine implicated in many autoimmune diseases. This increase in T_H_17 leads to a relative imbalance with the number of circulating T_regs_, contributing to immunotoxicity. In human trials of ipilimumab for melanoma, this increase in circulating T_H_17 cells was noted and associated with an increased risk of developing an irAE [3].

#### 2.2.2. Cross-Reactivity with Anti-Tumoral T Cells

The proliferation of effector T cells observed after CTLA-4 and PD-1 system blockades allows for strong anti-tumor T cells responses. However, there is concern for cross-reactivity between the tumor antigens and similar epitopes on healthy cells. For example, the highest rates of vitiligo irAEs were found amongst individuals treated for melanoma with ICIs, suggesting cross reactivity as a mechanism of irAEs, and in this instance, a reaction between activated anti-tumoral T cells and melanocytes [17]. Furthermore, shared TCRs between myocardium and tumor cells have been demonstrated in autopsies on fatal cases of myocarditis from ICIs [18].

The mechanisms involved in molecular mimicry have particular implications for neurological toxicities. For example, there are shared epitopes between myelin and melanocytes, in particular with multiple shared ganglioside antigens. This might explain why we see development of peripheral nerve demyelinating irAEs after ICI exposure [19,20]. In cases of ICI-induced autoimmune encephalitis, there are potential interactions between N-methyl-D-aspartic acid (NMDA) receptor subunits encoded by *GRIN2A* (Glutamate Ionotropic Receptor NMDA Type Subunit 2A), which is also commonly mutated in melanoma [19,21].

#### 2.2.3. Implications of PD-1 and PD-L1 Blockade

Unlike CTLA-4, which appears early on to facilitate downregulation of the T cell response, PD-1 appears several hours later and works differently in terms of its relationship with T_reg_ function and survival, as well as cytokine release. PD-1 binds both PD-L1 and PD-L2, which are present on a variety of cell types, including tumor cells, leading to downregulation of T cells. PD-1 blockade is associated with an increase in pro-inflammatory cytokine activity, such as increased circulating levels of interferon-gamma (IFNγ) and interleukin-10 (IL-10), which have positive implications for cancer treatment but can increase the risk of secondary irAEs [22].

Mouse models involving PD-1-deficient mice have shown significant autoimmunity, including inflammatory arthritis and lupus-like glomerulonephritis [23]. In humans, polymorphisms in the gene for PD-1, programmed death cell protein 1 (*PDCD1*), are associated with an increased risk of autoimmunity as well, including early-onset lupus [24].

#### 2.2.4. Role of Biomarker Analysis in ICI-Related ICIs

There are many challenges in developing biomarkers for ICI-related irAEs, as the mechanisms underlying irAEs are not well understood and far more complex than “traditional” cytotoxic chemotherapy-induced toxicities. As previously discussed, there are many different mechanisms involved, including global T_reg_ dysfunction, molecular mimicry, and activation of tissue-specific T cells that recognize antigens distinct from the tumor. Genetic, environmental, and patient-specific factors all likely play an additional important role in the pathophysiology of developing irAEs. Therefore, investigational biomarkers studies require detailed analysis of complex biological data. Furthermore, established predictive biomarkers for ICIs efficacy, including PD-L1 expression and tumor mutational burden (TMB), have not been shown to correlate with development of irAEs [25]. Thus, the optimal biomarkers for irAEs are not well defined yet.

Some of the already published research has explored cellular biomarkers, such as T cell repertoire, as a predictor of irAE development. Early diversification in the T cell repertoire induced by ipilimumab was seen in patients who had irAEs compared with those without irAEs. Interestingly, this increase in diversity occurred before the clinical manifestation of toxicity, suggesting that other steps might be required before development of irAEs [26].

There are also logistical hurdles in terms of clinicians being able to order useful biomarker tests in real-time in order to make clinical decisions, although various cytokine panels are becoming more accessible commercially, and as mentioned previously, certain cytokines and inflammatory factors are of particular interest to irAEs. In particular, IL-17 is one of the critical inflammatory cytokines that was found to be associated with a risk of developing severe immune-mediated diarrhea in melanoma patients treated with ipilimumab [27]. Elevated expression of 11 cytokines (granulocyte colony stimulating factor, granulocyte-macrophage colony stimulating factor (GM-CSF), fractalkine, fibroblast growth factor 2, IFN-α2, IL-12p70, IL-1a, IL-1b, IL-1 receptor antagonist (IL-1RA), IL-2, and IL-13) was found to be strongly associated with severe irAEs in melanoma patients. These cytokines were integrated into a single toxicity score and predicted toxicity in a validation cohort [28]. A second study assessed risk of developing irAEs by analyzing levels of 40 cytokines/chemokines in 65 cancer patients who were followed longitudinally. Those who developed irAEs had lower baseline levels of C-X-C motif chemokine ligand 9 (CXCL9), CXCL10, CXCL11, and CCL19 and a significantly higher fold increase (particularly for CXCL9 and CXCL10) at 2–3 weeks and 6 weeks post treatment [29].

The gut microbiome composition has also been implicated in development of irAEs and it is a potential biomarker. However, additional studies beyond characterization of the gut microbiota are needed [30,31,32]. Given the strong influence of genetic variation on autoimmunity and since the underlying mechanisms of toxicity due to ICIs are thought to be due to autoimmunity, there is a question of whether such variations impact the risk of developing irAEs. A number of *CTLA4* and *PD-l1* polymorphisms have been associated with autoimmune disorders such as rheumatoid arthritis and autoimmune endocrinopathies [33,34]. Genome-wide single nucleotide polymorphism (SNP) data from patients on ICIs has the potential to identify variants in the genome correlated with risk of irAEs [35]; although, a recently published study investigating selected SNPs in patients with irAEs from nivolumab showed no clinical implication in predicting toxicity. While this study has limitations and the authors emphasized future research embracing germline genetics, it demonstrates the challenges with developing biomarkers [36]. Genome-wide association studies (GWAS) have been used to identify risk loci for a variety of autoimmune diseases, indicating possible shared mechanisms, and may be of use going forward for predicting risk of irAEs [37].

### 2.3. Neurological Presentations of ICI-Related irAEs

A number of irAEs have been cited in the literature to date, with a great degree of heterogenicity in terms of where they occur and whether they are isolated events, recurrent, or even progressive. Neurological events tend to be higher-grade toxicity events, with exception of some types of peripheral neuropathy. Simultaneous irAEs in separate organ systems have also been noted, as well as sequential reactions with ICI rechallenging. Based on retrospective data, examples of non-neurological irAEs include new-onset inflammatory bowel disease [38], inflammatory arthritis [39], pneumonitis, and bullous dermatoses [13].

Neurological irAEs can involve the central, peripheral, and autonomic nervous systems. Peripheral neuropathy is commonly seen, but there are also many cases of myasthenia gravis, myelitis, and autoimmune encephalitis [8,9,10]. Early clinical trial data for ICIs found rates of immunological neurotoxicity for anti-CTLA4, anti-PD1, and a combination of anti-CTLA4 and PD1 of 3.8%, 6.1%, and 12.0%, respectively. The irAEs presented with a range of severities, from mild peripheral neuropathy to fulminant encephalitis and myasthenia gravis crisis [8].

#### 2.3.1. CNS Manifestations

##### Encephalopathy/Encephalitis

The clinical spectrum of irAEs from ICIs ranges from mild inattention to severely compromised mental status, seizures, and hallucinations [40]. A high index of suspicion is important given broad differences in patients with metastatic cancers, which may lead to diagnostic difficulty. Furthermore, while rare, some of these cases have been reported in the setting of positive antibodies associated with paraneoplastic syndromes, such as anti-Hu [41], anti-NMDAR [9], and anti-GAD-65 [42,43]. Median time to onset varies, with a median of 51.5 days and a range of 18–297 days [40].

##### Aseptic Meningitis

Patients present with symptoms of headache, photophobia, and neck stiffness. These symptoms are often associated with fever and vomiting [44,45]. Excluding infectious etiologies in cases suspicious for meningitis is imperative.

##### Demyelination Syndromes

Exacerbation or de novo development of CNS demyelination may occur with ICIs therapy, but they appear to be relatively uncommon, perhaps due to the practice of avoiding ICIs in people with preexisting conditions. In association with ipilimumab, two case reports of either severe relapse in a patient with stable multiple sclerosis (MS) or transition from radiologically isolated syndrome to clinically definite MS have been described in the literature [5,6]. A fatal MS relapse was described in a case report after a single dose of atezolizumab [7]. New onset CNS demyelination was reported in a patient who presented with subacute onset of confusion and multifocal white matter lesions consistent with tumefactive demyelination following ipilimumab and subsequently nivolumab treatments [46]. Another case described a patient with multifocal lesions in the splenium, frontal white matter, and optic nerve after ipilimumab therapy [47].

##### Vasculitis

Association between ICIs and CNS vasculitis has been reported particularly with ani-PD-1 [48,49]. A more recent systematic review demonstrated this association in 53 cases, of which 20 were confirmed by meeting all the inclusion criteria [50]. The study was not specific for vasculitis involving the nervous system; however, central and peripheral nervous system primary vasculitis was one of the most commonly identified types in addition to large vessel vasculitis. This review supports the experimental evidence that there is a link between the PD-1/PD-L1 axis and vasculitis [51].

##### Transverse Myelitis

Case reports of transverse myelitis in patients with melanoma who received anti-CTLA-4 or combination anti-CTLA-4 and anti-PD-1 have been described [52,53,54]. MRI findings varied from a focal T2 signal abnormality without expansion of the cord, to diffuse enhancement and a T2 hyperintense signal extending from the cervicomedullary junction down to the conus medullaris.

##### Neurosarcoidosis

While rare, there are two case reports of presumed sarcoidosis-like reaction involving the nervous system with the use of combination anti-CTLA-4 and anti-PD-1 therapy [55,56]. The development of neurologic symptoms in both cases was delayed (10–11 months following treatment). MRI showed leptomeningeal enhancement without evidence of metastasis on work-up.

##### Paraneoplastic Syndromes (PNS)

These conditions can create diagnostic dilemmas in terms of knowing whether a presentation is related to a paraneoplastic antibody, which might be representing disease recurrence, or is strictly induced by the ICI exposure. Limbic encephalitis, encephalomyelitis, basal ganglia encephalitis, enteric neuropathy, cerebellar ataxia, melanoma-associated retinopathy (MAR), and opsoclonus myoclonus syndrome are all described PNS associated with ICIs. The majority of patients received nivolumab either alone or in combination with ipilimumab. Positive serology to anti-CASPR2, anti-NMDAR, anti-CRMP5, anti-Ma2, anti-Hu, and multiple retinal antigens in a patient with MAR has been reported [57,58].

#### 2.3.2. PNS Manifestations

##### Neuropathies

Neuropathies are one of the most commonly reported adverse events across all organ system irAEs, with a wide spectrum of phenotypes [59,60,61]. They include axonal pure sensory or sensorimotor polyneuropathy, acute inflammatory demyelinating polyneuropathy (AIDP)-like syndrome, or a more protracted course, such as chronic inflammatory demyelinating polyneuropathy (CIDP), small fiber/autonomic neuropathy, mononeuritis multiplex, cranial neuropathies, polyradiculoneuropathies, neuralgic amyotrophy, and sensory neuronopathy. Presentations range from length-dependent sensory loss and asymmetric pain and weakness to diplopia and ptosis due to cranial nerve involvement. Patients with AIDP-like syndrome can progress rapidly and usually have symptom onset early within the first 3–4 ICI cycles [62]. The most commonly identified electrodiagnostic findings are abnormal motor conduction and demyelination, which can involve various segments of the peripheral nerves and may have significant heterogeneity among patients. Patients can have coexistent myopathy and neuromuscular junction dysfunction on electrophysiological studies [61]. It is important to note that in cases of AIDP-like illness, the classic albuminocytologic dissociation is not always identified [63].

##### Myasthenia Gravis (MG)

Myasthenia gravis commonly presents with generalized fatigue and bilateral fatigable ptosis, with other described symptoms including dyspnea, unilateral abducens nerve palsy, and myalgia. Bulbar symptoms are more common in ICI-associated MG than idiopathic MG [64]. De novo presentation is frequent in ICI-associated MG; in one review of 23 case reports, >70% of cases had no history of MG [65]. Patients can have associated myopathy, myositis, rhabdomyolysis, and myocarditis. Elevation of creatine kinase (CK) ranging from mild to high levels might occur more frequently in ICI-associated MG than ICI naïve patients, with one case series identifying high CK levels in over three quarters of patients [64,65,66]. Associated myopathy may lead to challenges in interpreting the electrodiagnostic studies in these cases. Antibodies for acetylcholine receptor may or may not be present [64,65]. To our knowledge, there have been no identified cases with muscle-specific tyrosine kinase (MuSK) antibodies.

### 2.4. Acute Management of ICI-Related irAE

Guidelines for irAE management have been suggested by the American Society of Clinical Oncology (ASCO) [67], the European Society of Medical Oncology (ESMO) [68], and the National Comprehensive Cancer Network (NCCN) [69] for identifying, grading, and managing immunotoxicity. General recommendations irrespective of the organ involvement include (1) highlighting the importance of patient and caregiver education on immunotherapies, including possible irAEs prior to initiating therapy and throughout the treatment course; (2) primary oncologists and consultants having a high level of suspicion that new symptoms may be treatment related; and (3) Grade I toxicities requiring close monitoring and evaluation, though Grade II–IV often warrant discontinuation of immunotherapy.

With regards to evaluation and treatment of neurological symptoms, clinicians should rule out direct neurological involvement or progression of cancer and identify other possible reversible causes. Neurological expertise is advised for all neurologic irAEs of Grade II or higher to guide evaluation and interpretation of neurological symptoms and signs, workup, and management. In our experiences, early involvement of neurological expertise has facilitated appropriate workup and management. Evaluation and testing should be guided by the neurological syndrome identified, and consists of verification of the neurological condition through a detailed history and neurological examination, ruling out alternative etiologies, and performance of ancillary studies such as neuroimaging, electromyography/nerve conduction study (EMG/NCS), and/or electroencephalogram (EEG). Cerebrospinal fluid (CSF) analysis is warranted when there is clinical suspicion of meningitis, encephalitis, myelitis, or GBS. CSF cytology should be performed in selected clinical syndromes.

ICIs causing mild (Grade I) neurologic symptoms may be continued under close observation. For Grade II or higher neurologic symptoms, checkpoint inhibitor therapy should be held until the nature of the irAE and symptom progression is defined. Depending on the neurological manifestation, for example all forms of transverse myelitis, the NCCN guidelines recommend permanent discontinuation of ICI [69]. For neurological toxicities of Grade II or higher, a corticosteroid equivalent of methylprednisolone 1 to 4 mg/kg should be initiated. Symptom control may require escalation of corticosteroid therapy to pulse-dose methylprednisolone (1 g daily for 5 days) in addition to intravenous immunoglobulin (IVIg) or therapeutic plasma exchange (PLEX). The decision to give IVIg or PLEX should be based on standard guidelines for treatment of the underlying neurological condition, access to treatment, and the impact of possible side effects on patients [69]. There are case reports of successful management of treatment-refractory irAEs from ICIs with agents such as rituximab, cyclophosphamide, and tocilizumab. Neurological symptoms should be monitored closely regardless of the toxicity grade as progression of symptoms can lead to significant morbidity and mortality (i.e., respiratory compromise, autonomic dysfunction, elevated intracranial pressure (ICP), and refractory seizures).

### 2.5. Restarting Immunotherapy after Toxicity

A number of management questions arise when a patient experiences an irAE from an ICI or when a patient with a preexisting autoimmune disease develops cancer potentially amenable to ICI treatment. Whether to continue concurrent disease-specific immunomodulators for long-term management of autoimmune diseases, such as multiple sclerosis, inflammatory bowel disease, and rheumatoid arthritis, while on ICI is a particular clinical dilemma as well. Halting disease-modifying treatment for autoimmune disease could lead to relapse, but continuing therapy might reduce the efficacy of the cancer immunotherapy or increase the risk of an irAE. Moreover, if an individual without preexisting autoimmune disease develops an irAE alongside successful control of their cancer, it remains to be understood whether the ICI might be restarted safely for any given individual [70].

## 3. Chimeric Antigen Receptor T Cell Therapies

### 3.1. Indications

CAR T cell therapies are a novel approach to cancer treatment with four currently approved agents in the United States [Table 2] and many others in the pipeline. They involve genetically engineering immune effectors cells, often autologous T cells, to express a CAR that recognizes tumor cell surface markers, such as CD-19. Upon encountering tumor Ag, the CAR T cell is activated, leading to cytokine secretion, T cell proliferation, and tumor cell lysis. The CAR itself consists of an extracellular tumor-targeting moiety (single-chain variable fragment derived from tumor-reactive monoclonal antibody) fused to one or more intracellular T cell signaling domains. Additional co-stimulatory domains might also be present.

In particular, this approach has shown great promise for hematological malignancies, including acute lymphoblastic leukemia, non-Hodgkin’s lymphomas, and multiple myeloma. Of note, preexisting brain metastases or primary brain malignancy are currently exclusionary criteria for most clinical trials and approved agents.

### 3.2. Mechanisms of Neurotoxicity from CAR T Cell Therapy

CAR T cell therapies are associated with development of both cytokine release syndrome (CRS) as well as neurotoxicity, more recently termed immune effector cell-associated neurotoxicity syndrome (ICANS).

As CAR T cells expand and engage tumor cells, CRS can develop typically within the first 1–14 days. CRS is the result of the cytokine surge associated with early raise in serum C-reactive protein (CRP), INFγ, IL-6, IL-10, and GM-CSF, among other pro-inflammatory cytokines.

ICANS is also associated with elevated cytokines in the blood, including INFγ, IL-6, and TNFα. It is thought that endothelial dysfunction leads to increased blood–brain barrier (BBB) permeability, allowing invasion of these cells into the central nervous system, precipitating neurotoxicity. Leakage of BBB has been demonstrated by an increase in CSF leukocyte and protein counts, as well as presence of CD4+ and CD8+ CAR T cells in the CSF. Elevated IL-2, granulocyte–macrophage colony-stimulating factor (GM-CSF), and ferritin are significantly associated with neurologic events of Grade 3 or higher. Moreover, autopsies of patients who died from CRS and ICANS have revealed a number of abnormalities, including microhemorrhages, parenchymal necrosis, and microinfarcts [71].

#### Biomarkers in CAR T Cell Therapy

Biomarkers for toxicity in CAR T cell therapy have been investigated in various studies. Based on the pathophysiological mechanisms underlying these toxicities, which include complex interactions between immune cells, tumor cells, cytokines, and chemokines, although the mechanisms of neurotoxicity are not well understood, inflammatory markers such as expression profiling of cytokines was studied. A study by Teachey et al. developed models that can predict severe CRS before patients become critically ill by measuring the cytokine and clinical biomarkers of 51 patients with acute lymphoblastic leukemia [72]. Other studies investigated cytokines in B cell acute lymphoblastic leukemia patients who developed neurotoxicity with severe toxicity, correlating to higher levels of cytokines [73]. Similarly, in an effort to identify biomarkers, these expression profiles were also investigated in chronic lymphocytic leukemia and diffuse large B cell lymphoma [74,75]. Additionally, high levels of CRP and ferritin have also been reported as predictive biomarkers [74]. Cellular biomarkers and biomarkers of endothelial activation are being investigated [76]. Overall, additional validation efforts are now needed to develop a more structured biomarker approach in this area from all of these observations.

### 3.3. Clinical Evaluation of Neurological Toxicity

Across clinical studies, there has been significant variation in grading of CRS and ICANS. ICANS, once thought to be a part of CRS and now considered to be a separate entity, was originally embedded in the CRS grading schema. Similar to CRS, ICANS can be applied to any immune effector cell (IEC) engaging therapy, not just CAR T cells. Adverse events associated with IEC engaging therapy may develop suddenly and progress rapidly to life-threatening events. Thus, establishing a baseline value according to standardized grading systems is essential.

The earliest CRS grading scale was the Common Terminology Criteria for Adverse Events (CTCAE, National Cancer Institute) [77]. The grading system has proven particularly challenging to define neurotoxicities given the variability in terminology and challenges with bedside applicability [78,79]. Lee and colleagues subsequently redefined the grading criteria for CRS around end-organ toxicities [80]. Memorial Sloan Kettering Cancer Center (MSKCC) criteria originally identified objective factors, including the serum cytokine levels, which is not easily applicable across clinical settings. An important development was a 10-point screening tool called the CARTOX-10 grading, which incorporated components of the Mini-Mental State Examination (MMSE), including alterations in speech, orientation, handwriting, and concentration. Another scoring system, the Immune Effector Cell-Associated Encephalopathy (ICE) score, includes an element for assessing the receptive aphasia seen in these patients. These screening tools were designed to overcome the subjectivity in grading many overlapping encephalopathy terms and remain widely used [81,82,83].

More recently, guidelines have been proposed by the American Society for Transplantation and Cellular Therapy (ASTCT). These expert guidelines have redefined terminology, with CRS defined as “a supraphysiologic response following any immune therapy that results in the activation or engagement of endogenous or infused T cells and/or other immune effector cells. Symptoms can be progressive, must include fever at the onset, and may include hypotension, capillary leak (hypoxia) and end organ dysfunction.” Grade 1 CRS is defined as fever (≥38.0 °C) with or without constitutional symptoms; Grade 2 CRS as fever (≥38.0 °C) with hypotension not requiring vasopressors and/or hypoxia requiring the use of oxygen delivered by a low-flow nasal cannula (≤6 L/min) or blow-by; Grade 3 CRS as fever (≥38.0 °C) with hypotension requiring one vasopressor with or without vasopressin and/or hypoxia requiring a high-flow nasal cannula (>6 L/min), facemask, nonrebreather mask, or venturi mask, not attributable to any other cause; and Grade 4 CRS as fever (≥38.0 °C) with hypotension requiring multiple vasopressors (excluding vasopressin) and/or hypoxia requiring positive pressure (e.g., CPAP, bilevel positive airway pressure, intubation, mechanical ventilation), not attributable to any other cause.

ASTCT defined ICANS as “a disorder characterized by a pathologic process involving the central nervous system following any immune therapy that results in the activation or engagement of endogenous or infused T cells and/or other immune effector cells. Symptoms or signs can be progressive and may include aphasia, altered level of consciousness, impairment of cognitive skills, motor weakness, seizures, and cerebral edema” [84]. Importantly, the ASTCT criteria exclude headache, tremor, myoclonus, asterixis, and hallucinations from the definition of neurotoxicity due to a lack of specificity. In the grading system, the final ICANS grade is determined by the most severe event among the different domains, based on the ICU scoring system. As per guidelines, mental status changes define the onset of ICANS after CAR T cell therapy. Importantly, there is separate grading criteria for pediatric patients.

#### Management of CRS and ICANS

The Society for Immunotherapy of Cancer (SITC) clinical practice guideline on IEC-related adverse events has recently advocated for the use of ASTCT criteria in monitoring for neurotoxicity [85]. In those patients who develop ICANS, recommendations include workup with CRP, complete metabolic panel, complete blood counts, fibrinogen, prothrombin time test, and international normalized ratio. A head CT is recommended, as well as consideration of EEG and brain MRI. Importantly, neuroimaging to evaluate for cerebral edema should not delay emergent management. Given that patients receive lymphodepleting chemotherapy ahead of CAR T cell infusion, clinicians must be cautious and exclude other causes of fever, hypotension, and hemodynamic instability, such as a systemic infection.

For adults with ASTCT Grade 2 CRS, or for elderly patient or those with extensive comorbidities, tocilizumab should be considered. If there is no improvement in CRS after one dose, then steroids can be added, and if no improvement after two doses of tocilizumab and steroids, other agents may be tried, including siltuximab, anakinra, and high-dose methylprednisolone [85].

For patients with ICANS Grade 2, steroids may be considered, but they are recommended for Grades 3 or 4. Steroid dosing still remains variable and poorly defined. To manage seizures, levetiracetam is recommended [85]. Studies have shown that tocilizumab may worsen neurotoxicity, with the proposed mechanism that IL-6 receptor blockade with tocilizumab may lead to increased circulating IL-6 in the CNS. Like with CRS, alternative monoclonal antibodies that have been used to manage neurotoxicity include siltuximab, a chimeric monoclonal antibody that directly binds IL-6, and Anakinra, a recombinant IL-1 receptor antagonist [4,86,87].

## 4. Future Research

With a variety of mechanisms now FDA approved and several others in the development pipeline, our experience with the efficacy and safety profile of such agents grows. While the current guideline suggestions provide the first steps towards treating irAEs, they lack a few critical components: (1) they are often relying on retrospective data as opposed to systematically gathered prospective data; and (2) they are often not based on immunobiological data that might lead to more individualized care.

We are still in need of prospective studies with both clinical and biomarker analysis to understand the various types of reactions. Novel technologies, such as single-cell RNA sequencing, are promising in identifying additional biomarkers. On the clinical side, randomized control trials for clinical management of irAEs are needed more than ever to help establish evidence-based treatment practices. Current ongoing trials include treatment beyond steroids with other biologics, such as tocilizumab and rituximab.

Furthermore, patients with known autoimmune diseases were excluded from clinical trials for ICIs over safety concerns; thus, other than case report data suggesting the potential for worsening of the underlying immunological diseases, large-scale prospective data have yet to be collected on this particular cohort. Ongoing trials assessing the safety of ICIs in patients with autoimmune disease are underway, with the hope for initial data in the upcoming year.

## 5. Conclusions

As treatment of cancer expands to harness the power of the human immune system, the need to understand irAEs and toxicity equally grows. Neurological toxicity is of particular concern given the often high grades of toxicity and lack of clinical trials to guide evidence-based management. Now that we have begun to describe the scope of the disease, we can begin to recognize these conditions earlier, to enhance the design of translational studies examining irAEs, and to develop clinical, radiological, and biomarkers with the hope of being able to treat patients with these conditions more safely and effectively in the near future.

## Figures and Tables

**Table 1:** FDA-approved immune checkpoint inhibitors and their specific clinical indications. Ipilimumab is the only FDA-approved anti-CTLA-4 monoclonal antibody. ALK, anaplastic lymphoma kinase. BRAF, B-Raf proto-oncogene. CTL-4, cytotoxic T lymphocyte-associated antigen 4. CRC, colorectal cancer. dMMR, mismatch repair deficient. EGFR, epidermal growth factor receptor. HCC, hepatocellular carcinoma. HNSCC, head and neck squamous cell carcinoma. MSI-H, microsatellite instability—high. mAb, monoclonal antibody. NSCLC, non-small cell lung cancer. PD-1, programmed cell death protein 1. PD-L1, programmed death ligand 1. RCC, renal cell carcinoma. SCLC, small cell lung cancer. SCC, squamous cell carcinoma. TMB-H, tumor mutational burden—high.

Drug	Target	Indications
Ipilimumab (Yervoy^®^)	CTLA-4	Melanoma, Unresectable or Metastatic
Pembrolizumab (Keytruda^®^)	PD-1	Melanoma, unresectable or metastatic Merkel cell carcinoma, recurrent locally advanced or metastatic CRC, unresectable or metastatic with MSI-H or dMMR NSCLC, no EGFR or ALK genomic alterations SCLC, metastatic Primary mediastinal large B-cell lymphoma, relapsed or refractory HNSCC Hodgkin Lymphoma, relapsed or refractory RCC, advanced (in combination with axitinib) Urothelial carcinoma, locally advanced or metastatic Gastric cancer, recurrent locally advanced or metastatic HCC, advanced Triple negative breast cancer *, locally recurrent, unresectable or metastatic (in combination with chemotherapy) Cervical cancer*, recurrent or metastatic cancer *, unresectable or metastatic with MSI-H or dMMR and no satisfactory alternative treatment options
Nivolumab (Opdivo^®^)	PD-1	Melanoma, unresectable or metastatic NSCLC, metastatic RCC, advanced Hodgkin Lymphoma, relapsed or progressed HNSCC Urothelial carcinoma, locally advanced or metastatic Esophageal squamous cell carcinoma, unresectable advanced, recurrent, or metastatic CRC*, metastatic with MSI-H or dMMR HCC *
Ipilimumab + Nivolumab (Yervoy^®^ + Opdivo^®^)	CTLA-4 + PD-1	Melanoma, unresectable or metastatic NSCLC, no EGFR or ALK genomic alterations RCC, intermediate or poor risk Malignant pleural mesothelioma, unresectable CRC *, metastatic with MSI-H or dMMR HCC *
Cemiplimab (Libtayo^®^)	PD-1	Cutaneous SCC, locally advance or metastatic HNSCC *, recurrent or metastatic CRC *, metastatic with MSI-H or dMMR HCC *
Durvalumab (Imfinzi^®^)	PD-L1	NSCLC, unresectable stage III SCLC, extensive stage (in combination with etoposide and either carboplatin or cisplatin)
Atezolizumab (Tecentriq^®^)	PD-L1	NSCLC, metastatic and no EGFR or ALK genomic alterations SCLC, extensive stage Melanoma, BRAF V600 mutation-positive unresectable or metastatic (in combination with cobimetinib and vemurafenib) Triple negative breast cancer, unresectable locally advanced or metastatic (in combination with protein-bound paclitaxel) Urothelial carcinoma, locally advanced or metastatic HCC, unresectable or metastatic (in combination with bevacizumab)
Avelumab (Bavencio^®^)	PD-L1	Merkel cell carcinoma, metastatic Urothelial carcinoma, locally advanced or metastatic RCC, advanced (in combination with axitinib)

* Continued FDA approval for this indication may be contingent upon verification and description of the clinical benefit in other confirmatory trials.

**Table 2:** FDA-approved CAR T cell therapies as of 10 March 2021. * R/R: refractory or relapsed. DLBCL: Diffuse large B-cell lymphoma. FL: Follicular lymphoma.

Generic Name	Brand	Indications
Axicabtagene ciloleucel	Yescarta^®^	R/R * large B-cell lymphoma (including DLBCL), primary mediastinal large B-cell lymphoma, high grade B-cell lymphoma, and DLBCL arising from FL
Tisagenlecleucel	Kymriah™	R/R B-cell precursor acute lymphoblastic leukemia (<25 years old), adult large B-cell lymphomas (includes DLBCL), high grade and DLBCL arising from FL
Brexucabtagene autoleucel	Tecartus^®^	Adults with R/R mantle cell lymphoma
Lisocabtagene maraleucel	Breyanzi^®^	R/R large B-cell lymphoma (including DLBCL not otherwise specified or arising from indolent lymphoma), high-grade B-cell lymphoma, primary mediastinal large B-cell lymphoma, FL grade 3B

## Data Availability

No new data were created or analyzed in this study. Data sharing is not applicable to this article.
